# An assessment of the psychometric properties of the Coping Strategies Questionnaire – Sickle Cell Disease (CSQ-SCD) among adults in the United States

**DOI:** 10.1186/s12955-024-02251-0

**Published:** 2024-04-22

**Authors:** Monika Salkar, Meagen Rosenthal, Kaustuv Bhattacharya, Sujith Ramachandran, Marie Barnard, John Young, John P. Bentley

**Affiliations:** 1 AbbVie Inc. Headquarters, 1 N. Waukegan Road, North Chicago, IL 60064 USA; 2 Department of Pharmacy Administration, University of Mississippi, University, MS USA; 3 Department of Psychology, University of Mississippi, University, MS USA

**Keywords:** Sickle cell disease, Psychometrics, Factor analysis, Coping

## Abstract

**Background:**

Previous studies have reported conflicting factor structures of the Coping Strategies Questionnaire - Sickle Cell Disease (CSQ-SCD). This study examined the psychometric properties of the CSQ-SCD among adults with SCD in the United States.

**Methods:**

This study implemented a cross-sectional study design with web-based self-administered surveys. Individuals with SCD were recruited via an online panel. Psychometric properties, including factorial and construct validity, and internal consistency reliability, of the CSQ-SCD were assessed.

**Results:**

A total of 196 adults with SCD completed the survey. Confirmatory factor analysis (CFA), using maximum likelihood estimation and the 13 subscale scores as factor indicators, supported a three-factor model for the CSQ-SCD compared to a two-factor model. Model fit statistics for the three-factor model were: Chi-square [df] = 227.084 [62]; CFI = 0.817; TLI = 0.770; RMSEA [90% CI] = 0.117 [0.101–0.133]; SRMR = 0.096. All standardized factor loadings (except for the subscales isolation, resting, taking fluids, and praying and hoping) were > 0.5 and statistically significant, indicating evidence of convergent validity. Correlations between all subscales (except praying and hoping) were lower than hypothesized; however, model testing revealed that the three latent factors, active coping, affective coping, and passive adherence coping were not perfectly correlated, suggesting discriminant validity. Internal consistency reliabilities for the active coping factor (α = 0.803) and affective coping factor (α = 0.787) were satisfactory, however, reliability was inadequate for the passive adherence coping factor (α = 0.531). Given this overall pattern of results, a follow-up exploratory factor analysis (EFA) was also conducted. The new factor structure extracted by EFA supported a three-factor structure (based on the results of a parallel analysis), wherein the subscale of praying and hoping loaded on the active coping factor.

**Conclusions:**

Overall, the CSQ-SCD was found to have less than adequate psychometric validity in our sample of adults with SCD. These results provide clarification around the conflicting factor structure results reported in the literature and demonstrate a need for the future development of a SCD specific coping instrument.

**Supplementary Information:**

The online version contains supplementary material available at 10.1186/s12955-024-02251-0.

## Introduction

Sickle cell disease (SCD) is a prevalent genetic disorder marked by chronic hemolytic anemia and recurrent painful vaso-occlusive crises (VOCs) [1, 2]. The frequency of VOC varies among patients, affecting their use of differing coping strategies. The relationship between coping and SCD management has been studied since the 1970s [3, 4]. Some patients face frequent episodes of pain, leading to hospitalizations and narcotic analgesia use, whereas others only have occasional pain crises [5]. Individuals with SCD differ in their abilities to cope with disease-related pain [6, 7].

Psychological coping responses, including behavioral and cognitive efforts to navigate stress, are linked to pain, adaptation, and health service utilization, even when adjusting for clinical indicators [1, 8, 9]. Gil et al. found that pain coping strategies significantly explained variance in pain severity, independent of demographics and disease severity [6]. While pain severity consistently predicts health service utilization [6, 8, 10–12], coping strategies, including negative thinking and passive adherence coping, are also associated with increased service utilization [7].

Recent studies using the Coping Strategies Questionnaire (CSQ) or the Coping Strategies Questionnaire-SCD (CSQ-SCD) [13–15] revealed distinct uses of coping strategies among adults with SCD as a function of opioid or alcohol use and perceptions of healthcare injustice. In the PiSCES study, while no significant differences were found in active or positive coping between opioid and non-opioid users, opioid users showed a higher prevalence of negative coping styles [15]. Alcohol users were more likely to employ coping mechanisms such as diverting attention and using self-statements compared to non-users [14]. Patients perceiving healthcare injustice were more likely to use catastrophizing and isolation coping measures [13]. Thus, coping has emerged as a crucial factor influencing the experiences of patients with SCD, affecting their quality of life.

In 1989, Gil et al. [6] developed the CSQ-SCD, the only SCD-specific tool for measuring typical approaches to coping with disease-related pain. The original analysis of the CSQ-SCD suggested two broad coping dimensions underlying the 13 subscales: ‘Coping attempts’ and ‘Negative thinking/passive adherence’ [6]. Subsequent analyses, however, yielded inconsistent results, particularly regarding the negative thinking/passive adherence factor [8, 16, 17]. This factor seems to merge two constructs: 1) negative cognitive/emotional/behavioral responses to SCD pain (e.g., catastrophizing), often viewed as maladaptive, and 2) adherence to clinicians’ recommended physiological pain management strategies (e.g., heat/cold/massage) [8, 16, 17]. These conceptual differences complicate interpretation of scores for this dimension (e.g., using a heating pad to cope with pain is not necessarily negative or maladaptive). Furthermore, relationships of this factor with other variables may be difficult to understand. Empirically, McCrae and Lumley [16] found that the subscales proposed to comprise the ‘Negative thinking/passive adherence’ dimension load on two separate factors. In examinations of the full CSQ-SCD, Anie et al. [8] reported three factors underlying the subscales (labeled as ‘active coping,’ ‘affective coping’, and ‘passive adherence coping’) as did McClish et al. [17].

Additionally, diverse methods have been used to evaluate the CSQ-SCD’s factor structure, including principal components analysis [16], exploratory factor analysis (EFA) [6], and what was described as higher-order factor analysis (which was actually an EFA of subscale scores as indicators) [8, 17]. To the best of our knowledge, a confirmatory factor analysis (CFA) on the subscale scores from the CSQ-SCD has not been reported in the literature. Furthermore, given the potential impact of different samples data on factor structure, periodic revalidation studies are crucial for factor-structure confirmation and addressing sampling-related concerns [18]. Thus, this study assessed the CSQ-SCD’s psychometric properties in a new sample of adult, US-based SCD patients, including convergent, discriminant, and factorial validity through CFA, along with internal consistency reliability.

## Methods

### Study design

The study employed a cross-sectional, web-based survey among a national convenience sample of US adults with SCD. All study procedures were approved by the University of Mississippi Institutional Review Board (Protocol #21x-130) under exempt status.

### Participants

The study sample included adults (≥ 18 years of age) with SCD, recruited with the help of Rare Patient Voice, a market research company that maintains a panel of SCD patients. Most of the patients in the panel have been recruited at SCD-related conferences and patient advocacy group meetings across the US. Given the nature of the statistical analysis plan for this study (i.e., CFA via structural equation modeling (SEM)), an a priori sample size of 200 patients with SCD was considered to be adequate [19]. Potential participants received an email outlining the purpose of the study, assuring confidentiality, and containing a Qualtrics survey link [20]. Respondents were provided a $15 Amazon gift card for participating.

### Measures

The survey instrument measured individual respondent demographics, clinical history, and coping strategies. The CSQ-SCD instrument was used to measure coping strategies. The CSQ-SCD consists of 13 subscales with 6 items per subscale: calming self-statements (CSS), diverting attention (DA), ignoring pain sensations (IPS), increasing behavioral activity (IBA), reinterpreting pain sensations (RPS), praying and hoping (PH), catastrophizing (CA), anger self-statements (AS), fear self-statements (FS), resting (RS), heat, cold, and massages (HCM), taking fluids (TF), and isolation (IS) [6]. Respondents rate the frequency of their use of each coping strategy when they feel pain from “0 = never do that” to “6 = always do that.” The instrument has been shown to have moderate to excellent internal consistency reliability (coefficient alphas ranging from 0.69 to 0.91 for the 13 subscales [6] and alphas of 0.80 and higher for the factors underlying the subscales [8]).

As has been done commonly in the psychometric studies outlined above, item responses were averaged to produce a mean for each subscale [6, 8, 16]. The exception to this general strategy was that if a subscale for a respondent had only a response to a single item, then the score for that subscale was set to missing. Two additional aspects were assessed by single items in the CSQ-SCD: perceived ability to control pain and perceived ability to decrease pain [6]. These two global items were not used in subsequent factor analyses, which is also consistent with previous studies [6, 8, 16].

The Adult Sickle Cell Quality of Life Measurement Information System (ASCQ-Me) pain episode measure and the ASCQ-Me medical history checklist were used to report the severity/frequency of pain events and the conditions related with SCD, respectively [21].

### Statistical analysis

Sample descriptive statistics were calculated in the form of frequencies and percentages of endorsements for categorical variables and means and standard deviations for continuous variables. Item-level analysis of the CSQ-SCD was conducted in terms of response frequencies as well as means and standard deviations (SD). When encountered, missing data were treated as a category with the number of subjects with missing responses represented as percentages. For CFA, the total available sample was used for the analysis rather than listwise deletion (i.e., full information maximum likelihood estimation, or FIML) [22]. Kurtosis and skewness coefficients were also calculated [19].

The CSQ-SCD’s factor structure was evaluated using CFA, an SEM technique used to assess the fit of a theoretically-constructed model. Two commonly tested models for CSQ-SCD include a two-factor, second-order model (i.e., using mean subscale scores as factor indicators) [6] and a three-factor, second-order factor model (i.e., using mean subscale scores as factor indicators) [8]. These models were previously explored by Gil et al. [6] and Anie et al. [8], with variations in extraction methods. In the current study, both models were tested using a process akin to higher-order CFA, where mean subscale scores served as factor indicators in a first-order CFA. Higher-order CFA is commonly thought of as consisting of the extraction of factors from first-order factors (this is an example of second-order CFA), which are themselves based on observed indicators (e.g., the items from a scale) [23]. However, Bagozzi and Edwards [24] note that first-order factor analysis of subscale scores – referred to as the partial aggregation model (which is what Gil et al. [6] and Anie et al. [8] actually did) – is conceptually equivalent to a second-order factor model (although not mathematically the same).

For the two-factor model [6], the DA, RPS, CSS, IPS, PH, and IBA subscales were specified to load on the coping attempts factor, while the CA, FS, AS, IS, TF, RS, and HCM subscales loaded on the negative thinking/passive adherence factor. The two factors were allowed to correlate. In the three-factor model [8], the IPS, CSS, IBA, DA, and RPS subscales were specified to load on the active coping factor, the CA, AS, FS, PH, and IS subscales on the affective coping factor, and the RS, TF, and HCM subscales on the passive adherence coping factor. Factors were all allowed to intercorrelate. Due to the continuous nature of CSQ-SCD subscale scores, maximum likelihood estimation (MLE) was used [23]. Higher-order CFA using the individual items was not reported due to estimation problems (i.e., nonconvergence). All CFA models were estimated using Mplus version 8.4 [25]. The following five fit statistics were assessed for each model: χ2 statistic, the root mean square error of approximation (RMSEA), the Tucker Lewis Index (TLI), the comparative fit index (CFI), and the standardized root mean square residual (SRMR). Bagozzi and Yi [26] suggest that for a well-fitting model, the RMSEA, TLI, CFI should be ≤ 0.08, ≥ 0.92, and ≥ 0.93, respectively. For a good fitting model, SRMR should be less than 0.08 [27].

Factor loadings, average variance extracted (AVE), and correlations among the 13 subscales were used to estimate convergent validity of the CSQ-SCD. Standardized factor loadings of 0.5 or higher were considered evidence of adequate validity [28]. Likewise, AVE values of 0.5 or greater were suggestive of adequate convergence [28]. Pearson’s correlations were categorized as small (0.1–0.29), moderate (0.3–0.49), or strong (≥ 0.5) [29]. Subscales comprising each latent trait in the model tested were hypothesized to correlate strongly.

Discriminant validity was assessed using three methods. First, the fit of the best fitting model was compared to that of a similar model where the latent factor correlations (e.g., for the three-factor model, the correlations between active coping, affective coping, and passive adherence coping) were fixed to 1 (latent construct discriminant validity). This test was carried out using the MODEL TEST option in Mplus [23, 25]. A significant difference in the model fit (Wald’s χ2 statistic) was suggestive of discriminant validity [30]. Second, AVEs for each latent variable were calculated and compared to the square of correlation estimates between all possible latent variables. If both AVEs for a given pairwise comparison exceeded the sum of the squared correlation value, this suggested that the latent construct explained a greater proportion of the variance in its indicator items than did another latent construct, indicating discriminant validity [31]. Third, correlations ≤ 0.40 between subscale scores underlying one factor with subscale scores underlying separate factors were indicative of discriminant validity. For example, for the three-factor model, weak correlations of the subscales IPS, CSS, IBA, DA, and RPS with the subscales underlying affective coping (i.e., CA, AS, FS, PH, and IS subscales) and passive adherence coping (i.e., RS, TF and HCM subscales) were hypothesized.

To assess the reliability for the CSQ-SCD, Cronbach’s alpha (α) and McDonald’s omega were calculated for the active coping, affective coping, and the passive adherence coping factors. Values ≥ 0.70 were suggestive of adequate internal consistency reliability, with values ≥ 0.80 considered preferable [32].

## Results

### Sample characteristics

The final sample consisted of 196 adults with SCD (Table 1). The majority of the participants were female (76.50%), Black (83.67%), and suffered from more than two medical health conditions (58.16%). Participants had an average of three sickle cell pain attacks in the past year and the most common duration for these attacks was 1–3 days (32.65%). The sickle cell pain severity (mean = 51.95, SD = 8.10) and frequency scores (mean = 51.41, SD = 9.51) were similar to the average score of 50 (for both severity and frequency) in the reference population [33].

**Table 1:** Demographic and clinical characteristics of the sample (*N* = 196)

Characteristics	N (%)
**SCD type**	
Hemoglobin SS (SCA)	135 (68.88)
Other*	61 (31.12)
Age, mean (SD)	36 (9.88)
**Sex**	
Male	27 (13.78)
Female	150 (76.50)
Missing	19 (9.70)
**Race/ethnicity**	
African American/Black	164 (83.67)
Other^++^	13 (6.63)
Missing	19 (9.69)
**Living status**	
Living alone	35 (17.86)
Living with someone	141 (71.94)
Missing	20 (10.20)
**Education level**	
High school or less	30 (15.31)
More than high school	147 (75.00)
Missing	19 (9.69)
**Employment status**	
Employed (full time/part-time)	80 (40.82)
Unemployed	97 (49.49)
Missing	19 (9.69)
**Region**	
Northeast	46 (23.47)
Midwest	39 (19.89)
South	69 (35.20)
West	23 (11.73)
Missing	19 (9.69)
**Insurance**	
Yes	171 (87.24)
No	5 (2.55)
Missing	20 (10.20)
**Insurance type**	
Public	106 (54.08)
Private	46 (23.47)
Both	19 (9.69)
Missing	24 (12.24)
**Access to opioids**	
Most of the times	30 (15.31)
Sometimes	84 (42.86)
Never	58 (26.60)
Missing	24 (12.24)
**Medical health conditions** ^**^**^	
At least 2	82 (41.84)
More than 2	114 (58.16)
**Duration of most recent pain attack (crisis)**	
1–23 h	35 (17.86)
1–3 days	64 (32.65)
4–6 days	36 (18.37)
1–2 weeks	31 (15.82)
More than 2 weeks	13 (6.63)
Missing	17 (8.67)
**Number of sickle cell pain attacks (crises) in the past year, mean (SD)**	3.12 (1.22)
**Sickle cell severity score**^**%**^, **mean (SD)**	51.95 (8.10)
**Sickle cell frequency score**^**%**^, **mean (SD)**	51.41 (9.51)

SCA, Sickle Cell Anemia; SD, Standard Deviation; QOL, Quality of Life

*Hemoglobin SC, Hemoglobin S beta-thalassemia (zero), Hemoglobin S beta-thalassemia (plus), Hemoglobin SD

^++^Other includes White/Caucasian, American Indian/Alaskan Native, Asian, Native Hawaiian/Other Pacific Islander, Hispanic, Middle Eastern, Mixed race

^^^Measured using the ASCQ-Me Medical History Checklist

^%^Measured using the ASCQ-Me Pain Episode Measure

### Verification of the factor structure of the CSQ-SCD using CFA

**Fig. 1:**
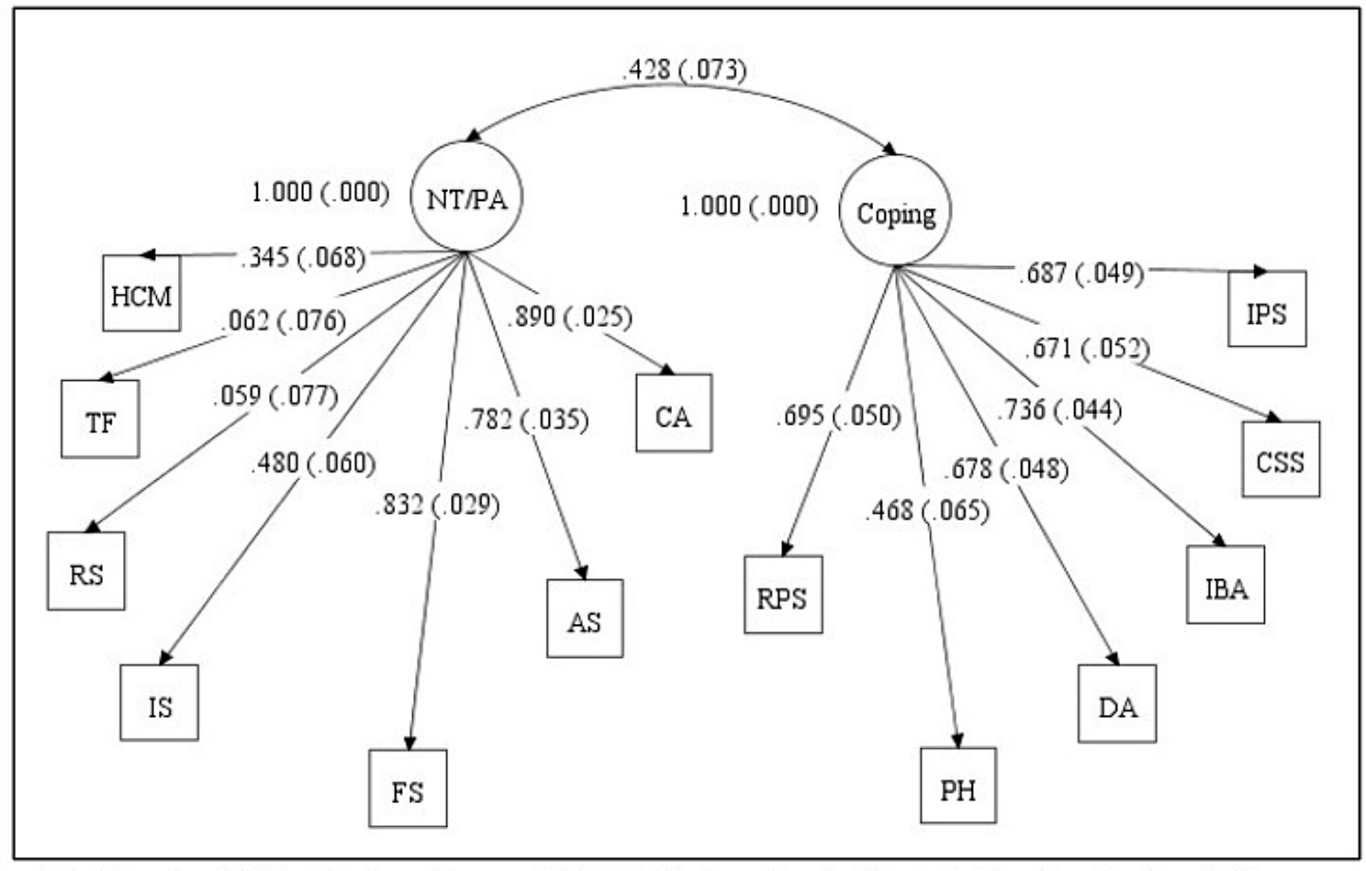
Two-factor model of CSQ-SCD based on Gil et al. [6] depicting standardized factor loadings. *Note* Negative thinking/Passive adherence (NT/PA), Coping attempts (Coping), Diverting attention (DA), Reinterpreting pain sensations (RPS), Calming self-statements (CSS), Ignoring pain sensations (IPS), Increasing behavioral activity (IBA), Praying and hoping (PH), Catastrophizing (CA), Fear self-statements (FS), Anger self-statements (AS), Isolation (IS), Resting (RS), Taking fluids (TF), Heat/cold/massage (HCM)

**Fig. 2:**
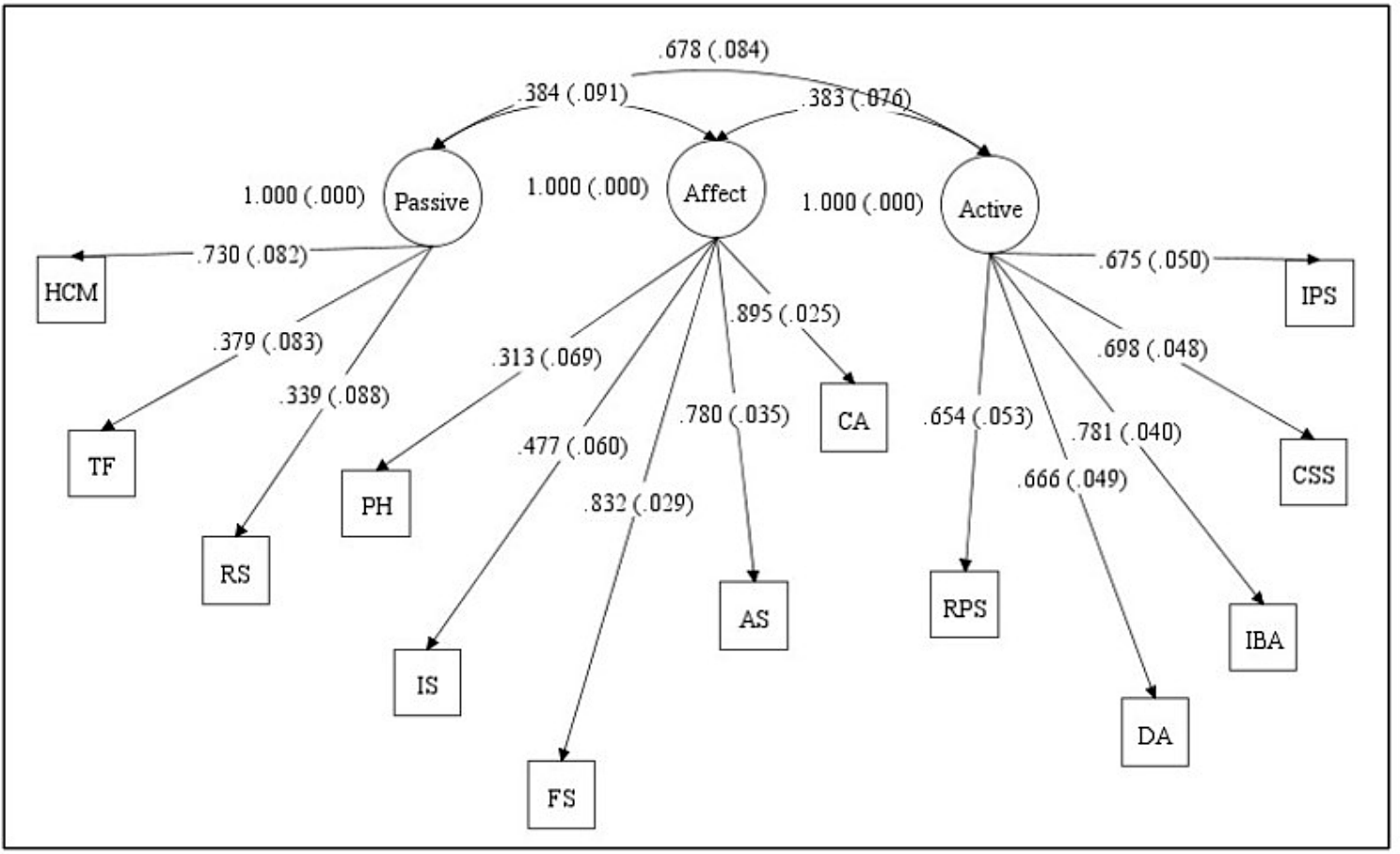
Three-factor model of CSQ-SCD based on Anie et al. [8] depicting standardized factor loadings. *Note* Passive adherence coping (Passive), Affective coping (Affective), Active coping (Active), Diverting attention (DA), Reinterpreting pain sensations (RPS), Calming self-statements (CSS), Ignoring pain sensations (IPS), Increasing behavioral activity (IBA), Praying and hoping (PH), Catastrophizing (CA), Fear self-statements (FS), Anger self-statements (AS), Isolation (IS), Resting (RS), Taking fluids (TF), Heat/cold/massage (HCM)

Supplementary Table S1 shows the mean scores, Cronbach’s alphas, as well as skewness and kurtosis coefficients for the 13 subscales of the CSQ-SCD. The skewness and kurtosis coefficients for all the CSQ-SCD subscales were found to be within a range of -1.094 and 0.889, indicating normally distributed data. The two measurement models tested to examine the factorial validity of the CSQ-SCD can be found in Figs. 1 and 2. The two-factor model had poor fit, and although the three-factor model fit better, its fit was nevertheless inadequate (Table 2). Modification indices for the three-factor model suggested that allowing the praying and hoping (PH) subscale to load on the passive adherence coping factor instead of the affective coping factor would significantly improve model fit. This improved fitting model still had less than satisfactory fit indices (Chi-square [df] = 197.881 [62]; CFI = 0.850; TLI = 0.811; RMSEA [90% CI] = 0.106 [0.089–0.122]; SRMR = 0.074).

**Table 2:** Summary of model fit indices for the CSQ-SCD confirmatory factor models

Fit Statistics	Model 1	Model 2
Chi-square (df)	266.154 (64)	227.084 (62)
CFI	0.776	0.817
TLI	0.727	0.770
RMSEA (90% CI)	0.127 (0.111–0.143)	0.117 (0.101–0.133)
SRMR	0.112	0.096

Model 1 – CSQ-SCD CFA model based on Gil et al. [6] (two-factor model)

Model 2 – CSQ-SCD CFA model based on Anie et al. [8] (three-factor model)

Note: df, degrees of freedom; CFI, Comparative Fit Index; TLI, Tucker-Lewis Index; RMSEA, Root Mean Square Error of Approximation; SRMR, Standardized Root Mean Square Residual; CI, Confidence Interval

### Evaluation of the convergent and discriminant validity of the CSQ-SCD

Given the somewhat better fit of the three-factor model, evaluation of convergent validity, discriminant validity, and internal consistency reliability focused on that model. All standardized factor loadings were statistically significant at α = 0.05 and all met or exceeded the 0.5 criteria for factor loadings except for the subscales PH, RS, and TF (Table 3). The AVE for active coping was 0.485, for affective coping was 0.485, and for passive adherence coping was 0.264. Supplementary Table S2 depicts the correlation matrix for the 13 CSQ-SCD subscales. Correlations between subscales underlying the same latent factor were moderate to weak. Subscales underlying the active coping factor, namely DA, IPS, CSS, IBA, and RPS had moderate correlations with each other compared to the subscales underlying the affective coping and passive adherence coping factors. Although the subscales AS, FS, IS, and CA from the affective coping factor were moderately-to-strongly correlated with each other, the praying and hoping (PH) subscale had small correlations with these subscales. In addition, for the passive adherence coping factor, the subscale heat/cold/massage had small correlations with the resting and taking fluids subscales. Overall, the standardized factor loadings, AVE for each factor, and subscale correlations provide some evidence of convergent validity for the CSQ-SCD.

**Table 3:** Standardized factor loadings for the final three-factor model of coping for the CSQ-SCD among adults with SCD

Subscales	Estimate^^^ (SE)
**Latent factor: active coping**	
Diverting attention (DA)	0.666 (0.049)
Reinterpreting pain sensations (RPS)	0.654 (0.654)
Calming self-statements (CSS)	0.698 (0.048)
Ignoring pain sensations (IPS)	0.675 (0.050)
Increasing behavioral activity (IBA)	0.781 (0.040)
**Latent factor: affective coping**	
Praying and hoping (PH)	0.313 (0.069)
Catastrophizing (CA)	0.895 (0.025)
Fear self-statements (FS)	0.832 (0.029)
Anger self-statements (AS)	0.780 (0.035)
Isolation (IS)	0.477 (0.060)
**Latent factor: passive adherence coping**	
Resting (RS)	0.339 (0.088)
Taking fluids (TF)	0.379 (0.083)
Heat/cold/massage (HCM)	0.730 (0.082)
**Latent factor correlations**	
Latent active coping factor with latent affective coping factor	0.383 (0.076)
Latent active coping factor with latent passive adherence coping factor	0.678 (0.084)
Latent affective coping factor with latent passive adherence coping factor	0.384 (0.091)

^^^All factor loadings were significant at α = 0.05

Three separate methods were employed to assess the discriminant validity of CSQ-SCD. First, correlations between each pair of factors were fixed to 1, one at a time, yielding significant Wald’s chi-square values, suggesting adequate discriminant validity (Table 4) [30]. Second, the difference between the AVE for each latent factor and the square of each latent factor correlation was found to be positive in most comparisons (Table 5), providing some evidence of discriminant validity [31]. Last, subscales comprising the active coping, affective coping, and passive adherence coping factors had weak to moderate correlation with subscales not underlying their own factor (Supplementary Table S2). However, the subscale praying and hoping (PH) from the affective coping factor was moderately correlated with the subscales DA and RPS underlying the active coping factor and the subscale HCM underlying the passive adherence coping factor. Overall, the CSQ-SCD was found to have moderately acceptable discriminant validity.

**Table 4:** Discriminant validity (i.e., tests of perfect correlations) assessed by Wald’s Chi-square test

Model^a^ (correlation fixed to 1)	Wald’s Chi-square Value	DF	P-value
Active coping with affective coping	66.264	1	< 0.001
Affective coping with passive adherence coping	45.958	1	< 0.001
Active coping with passive adherence coping	14.858	1	< 0.001

^a^Model was compared to base model with no restrictions on correlation between factors – i.e., correlations were freely estimated; significant tests indicate that the factors are not perfectly correlated, providing evidence of discriminant validity. DF, degrees of freedom

**Table 5:** Discriminant validity assessed by evaluating the difference between the AVE for each latent factor and the square of the latent factor correlation

Squared correlations	AVEs	Discriminant validity
Latent active coping factor with latent affective coping factor = 0.147	Latent active coping factor = 0.485	0.485 − 0.147 = 0.338 0.485 − 0.147 = 0.338
Latent active coping factor with latent passive adherence coping factor = 0.460	Latent affective coping factor = 0.485	0.485 − 0.460 = 0.025 0.264–0.460 = −0.196
Latent affective coping factor with latent passive adherence coping factor = 0.147	Latent passive adherence coping factor = 0.264	0.485 − 0.147 = 0.338 0.264 − 0.147 = 0.117

AVE, Average variance extracted

Reliability (Cronbach’s alpha and McDonald’s omega) for two of the CSQ-SCD factors was satisfactory (i.e., active coping and affective coping) and inadequate for passive adherence coping (Table 6).

**Table 6:** Reliability analysis for the CSQ-SCD components among adults with SCD

Factor	Cronbach’s alpha	McDonald’s omega	Mean	No. of subscales
Entire scale	0.817	0.803	3.413	13
Active coping	0.803	0.803	2.880	5
Affective coping	0.787	0.822	3.343	5
Passive adherence coping	0.531	0.552	4.404	3

### Re-assessment of the factor structure of the CSQ-SCD through EFA

CFAs for both models showed inadequate fit, prompting an EFA for further exploration of the factor structure of the CSQ-SCD. As in previous studies [6, 8, 16, 17], mean subscale scores were used as factor indicators and factors were extracted using the principal factors method. The Kaiser-Meyer-Olkin measure of sampling adequacy was 0.78 and the Bartlett’s test of sphericity was significant (*p* < 0.001), indicating that EFA could be applied.

The number of factors was decided based on the scree-plot, the cumulative variance explained, the interpretability of factor loadings, and parallel analysis. Four eigenvalues were greater than 1, while both the parallel analysis and scree plot suggested three factors (Supplementary Fig. S1). The percentage of cumulative variance explained by the extracted three factors and four factors was 58% and 67%, respectively. Based on the interpretability of factor loadings (Table 7), a three-factor EFA model was deemed to be more appropriate compared to the four-factor model.

Following factor extraction, varimax rotation was used to aid in the interpretation of the EFA results. The EFA factor structure was similar to that reported by Anie et al. [8], except that in the current study’s analysis the subscales of PH and HCM loaded highest on the active coping factor rather than the affective coping and passive adherence coping factors, respectively.

**Table 7:** Varimax rotated factor loadings for three-factor exploratory factor analysis model

Coping strategy	Factor 1	Factor 2	Factor 3
**Active coping**			
Diverting attention (DA)	0.522		
Reinterpreting pain sensations (RPS)	0.625		
Calming self-statements (CSS)	0.647		
Ignoring pain sensations (IPS)	0.741		
Increasing behavioral activity (IBA)	0.734		
Praying and hoping (PH)	0.332		
Heat/cold/massage (HCM)	0.381		
**Affective coping**			
Catastrophizing (CA)		0.892	
Fear self-statements (FS)		0.800	
Anger self-statements (AS)		0.788	
Isolation (IS)		0.451	
**Passive adherence coping**			
Resting (RS)			0.532
Taking fluids (TF)			0.477

Subscales were allowed to load on all factors; only the highest loading for each subscale is reported in this table (other loadings were suppressed to aid interpretation)

## Discussion

As new pain management strategies for SCD evolve, a psychometrically-sound, SCD-specific coping instrument is crucial. Such a tool would help to advance coping research, aiding healthcare providers in understanding diverse coping strategies. This insight can drive new effective methods and wider dissemination for enhanced support, through patient advocacy, routine medical appointments, or mental health therapy.

The current study assessed the validity (factorial, convergent, and discriminant) and internal consistency reliability of the CSQ-SCD among adults. CFA was used to examine model fit of previously extracted CSQ-SCD factor structures. Based on model fit indices, the factorial validity of the three-factor model based on Anie et al. [8] had a relatively better fit compared to the two-factor model from Gil et al. [6], although neither fit was adequate in terms of published standards for interpretation. Still, the general pattern of results was consistent with a previous study conducted by McCrae and Lumley [16], wherein the factor analysis of the seven subscales of ‘Negative thinking/passive adherence’ loaded on two separate factors, indicating a three-factor structure of the CSQ-SCD.

The CSQ-SCD showed some evidence of convergent and discriminant validity though not very strong evidence across all the tests employed. The internal consistency reliability of the active and affective coping factors was good but was low for the passive adherence coping factor. Given the factorial validity findings, an EFA was conducted for further “exploration” of the poor-fitting CFA models [18]. The EFA results suggested that the praying and hoping and the heat/cold/massage subscales loaded highest on the active coping factor, findings that were different from past analyses of the CSQ-SCD. However, the loadings for these subscales are below acceptable standards for practical significance [28], suggesting that the somewhat poor performance of the CSQ-SCD with respect to factorial validity may be related to these subscales. Interestingly, modification indices from the CFA suggested that allowing the praying and hoping subscale to load on the passive adherence coping factor would improve model fit. All of this suggests that there does not appear to be a clear role for the praying and hoping subscale in the factor structure of the CSQ-SCD.

Different from the current study, Anie et al.’s model was constructed with a dataset from SCD patients in London who visited the hospital to consult with a clinician regarding their health without any strict exclusion or inclusion criterion [8]. Thus, it was possible for healthy patients taking an active role in their healthcare to participate in the study. Additionally, Anie et al. used a relatively smaller sample size of 96 patients [8]. These differences may have resulted in differences in the factor structure.

The current study had some limitations. The cross-sectional study design prevented predictive validity or test-retest reliability assessment of the CSQ-SCD. Thus, future studies should include longitudinal data. Participants’ relatively good physical functioning could limit generalizability of the findings. Assessing measurement invariance of coping measures among different groups comprising the SCD patient population is an important avenue for future research. Although the current study used a sample size larger than most previously published reports, the sample is nevertheless considered somewhat small for the use of CFA via SEM. Given that the population being studied is somewhat limited in size, our sample of 196 may still be considered acceptable [19].

This was the first US-based study to conduct a CFA of the CSQ-SCD among adults with SCD. In addition, previous published reports examining the CSQ-SCD factor structure have employed smaller sample sizes or conducted research only in Black patients. The CSQ-SCD instrument was developed in 1989 when there were limited treatment options for SCD. With treatment advancements and improved life expectancy, patients can now better manage their disease and may have devised different coping strategies. In addition, much has changed regarding the methods used for the development of patient-reported outcomes since 1989. Overall, this study’s findings emphasize the need for the development of a new concise and robust SCD-specific coping instrument. To develop this instrument effectively, qualitative research methods such as cognitive interviews and focus groups with SCD patients are needed to better understand patients’ perspectives. In addition, research efforts in this area may also require examining the theoretical underpinnings of coping strategies used by individuals with sickle cell disease [34]. Iterative development of the instrument via collaboration with experts in psychometrics, clinical experts, and patients and their advocates is essential for deriving a more psychometrically-sound instrument that can be incorporated into health policy and clinical decision-making studies.

## Conclusion

This study adds to existing evidence regarding the unsatisfactory psychometric properties of the CSQ-SCD instrument and provides clarification around the conflicting factor structure results reported in the literature. The scale demonstrated poor factorial and mediocre convergent and discriminant validity. In addition, the scale had poor internal consistency reliability for the passive adherence coping factor. In summary, the study findings provide a basis for future development of SCD-specific coping instruments and leveraging the information on the coping strategies employed by patients into providing appropriate psychosocial support.

### Electronic supplementary material

Below is the link to the electronic supplementary material.

Supplementary Material 1

## Data Availability

The datasets used and/or analyzed during the current study are available from the corresponding author on reasonable request. In addition, in the supplementary information, the authors have provided the results of item-level confirmatory factor analyses (CFAs) for the 13 subscales of the CSQ-SCD, correlation matrices (Pearson and polychoric correlations) and means/standard deviations for the 78 items of the CSQ-SCD, and the Mplus and SPSS code used to generate certain results discussed within the article. As suggested by an anonymous reviewer, these item-level results may help pinpoint problematic items comprising the subscales.

## Abbreviations

AS: Anger Self-Statements
AVE: Average Variance Extracted
CA: Catastrophizing
CFA: Confirmatory Factor Analysis
CFI: Comparative Fit Index
CI: Confidence Interval
CSQ-SCD: Coping Strategies Questionnaire – Sickle Cell Disease
CSS: Calming Self-Statements
DA: Diverting Attention
DF: Degrees of Freedom
EFA: Exploratory Factor Analysis
FIML: Full Information Maximum Likelihood Estimation
FS: Fear Self-Statements
HCM: Heat Cold Massage
IBA: Increasing Behavioral Activity
IPS: Ignoring Pain Sensations
IS: Isolation
MLE: Maximum Likelihood Estimation
PH: Praying and Hoping
QOL: Quality of Life
RMSEA: Root Mean Square Error of Approximation
RPS: Reinterpreting Pain Sensations
RS: Resting
SCA: Sickle Cell Anemia
SCD: Sickle Cell Disease
SD: Standard Deviation
SEM: Structural Equation Modeling
SRMR: Standardized Root Mean Square Residual
TF: Taking Fluids
TLI: Tucker Lewis Index
UK: United Kingdom
US: United States
VOC: Vaso-Occlusive Crises
