# Supplementary material for: An assessment of the psychometric properties of the Coping Strategies Questionnaire – Sickle Cell Disease (CSQ-SCD) among adults in the United States
Source: Health Qual Life Outcomes. 2024 Apr 22;22:35. doi: 10.1186/s12955-024-02251-0 (PMC11034024; doi:10.1186/s12955-024-02251-0)
Supplement: Supplementary file 1 — Supplementary Material 1 [file 12955_2024_2251_MOESM1_ESM.docx]

**Supplementary Information**

Supplementary Table S1: Subscale-level characteristics for the CSQ-SCD among adults with SCD

Supplementary Table S2: Subscale correlations for the CSQ-SCD

Supplementary Figure S1: Scree plot and parallel analysis based on sample eigenvalues

Supplementary Table S3: Standardized factor loadings and fit statistics from item-level CFAs of the 13 subscales for the CSQ-SCD (Tables S3a – S3m)

Supplementary Table S4: Item means, standard deviations, and correlations (Pearson and polychoric) for the CSQ-SCD (Tables S4a – S4c)

MPlus and SPSS code

**Supplementary Table S1: Subscale-level characteristics for the CSQ-SCD among adults with SCD**

|  | N | Missing | Mean (SD) | Skewness | | Kurtosis | | Cronbach’s Alpha |
| --- | --- | --- | --- | --- | --- | --- | --- | --- |
|  |  |  |  | Statistic | Std. error | Statistic | Std. error |  |
| Diverting attention (DA) | 196 | 0 | 3.362 (1.370) | -0.196 | 0.174 | -0.587 | 0.346 | 0.813 |
| Reinterpreting pain sensations (RPS) | 193 | 3 | 1.442 (1.269) | 0.889 | 0.175 | 0.399 | 0.348 | 0.842 |
| Calming self-statements (CSS) | 193 | 3 | 4.134 (1.063) | -0.198 | 0.175 | -0.588 | 0.348 | 0.760 |
| Ignoring pain sensations (IPS) | 192 | 4 | 2.421 (1.161) | 0.128 | 0.175 | -0.361 | 0.349 | 0.741 |
| Increasing behavioral activity (IBA) | 195 | 1 | 3.081 (1.014) | 0.164 | 0.174 | -0.094 | 0.346 | 0.557 |
| Praying and hoping (PH) | 194 | 2 | 4.134 (1.097) | -0.693 | 0.175 | 0.842 | 0.347 | 0.627 |
| Catastrophizing (CA) | 194 | 2 | 2.809 (1.380) | 0.226 | 0.175 | -0.634 | 0.347 | 0.849 |
| Fear self-statements (FS) | 193 | 3 | 3.237 (1.311) | 0.111 | 0.175 | -0.540 | 0.348 | 0.824 |
| Anger self-statements (AS) | 196 | 0 | 3.024 (1.224) | -0.118 | 0.174 | -0.663 | 0.346 | 0.766 |
| Isolation (IS) | 196 | 0 | 3.491 (1.262) | -0.254 | 0.174 | -0.290 | 0.346 | 0.829 |
| Taking fluids (TF) | 196 | 0 | 4.732 (1.072) | -1.094 | 0.174 | 1.396 | 0.346 | 0.792 |
| Resting (RS) | 194 | 2 | 4.437 (0.980) | -0.830 | 0.175 | 1.324 | 0.347 | 0.712 |
| Heat/cold/massage (HCM) | 196 | 0 | 4.057 (1.002) | -0.581 | 0.174 | 0.128 | 0.346 | 0.634 |

**Supplementary Table S2: Subscale correlations for the CSQ-SCD**

|  | **DA** | **RPS** | **CSS** | **IPS** | **PH** | **CA** | **FS** | **AS** | **IBA** | **IS** | **TF** | **RS** | **HCM** |
| --- | --- | --- | --- | --- | --- | --- | --- | --- | --- | --- | --- | --- | --- |
| **DA** | 1 | .449^**^ | .432^**^ | .335^**^ | .440^**^ | .356^**^ | .344^**^ | .250^**^ | .517^**^ | .279^**^ | .216^**^ | .251^**^ | .382^**^ |
| **RPS** | .449^**^ | 1 | .319^**^ | .587^**^ | .361^**^ | .403^**^ | .309^**^ | .325^**^ | .453^**^ | .260^**^ | .094 | .074 | .302^**^ |
| **CSS** | .432^**^ | .319^**^ | 1 | .493^**^ | .291^**^ | .110 | .172^*^ | .183^*^ | .586^**^ | .150^*^ | .191^**^ | .187^**^ | .337^**^ |
| **IPS** | .335^**^ | .587^**^ | .493^**^ | 1 | .193^**^ | .206^**^ | .120 | .243^**^ | .485^**^ | .153^*^ | .023 | .024 | .216^**^ |
| **PH** | .440^**^ | .361^**^ | .291^**^ | .193^**^ | 1 | .287^**^ | .252^**^ | .190^**^ | .244^**^ | .052 | .102 | .230^**^ | .367^**^ |
| **CA** | .356^**^ | .403^**^ | .110 | .206^**^ | .287^**^ | 1 | .758^**^ | .689^**^ | .172^*^ | .395^**^ | -.009 | -.010 | .292^**^ |
| **FS** | .344^**^ | .309^**^ | .172^*^ | .120 | .252^**^ | .758^**^ | 1 | .643^**^ | .115 | .338^**^ | .087 | .057 | .292^**^ |
| **AS** | .250^**^ | .325^**^ | .183^*^ | .243^**^ | .190^**^ | .689^**^ | .643^**^ | 1 | .118 | .504^**^ | .047 | .017 | .236^**^ |
| **IBA** | .517^**^ | .453^**^ | .586^**^ | .485^**^ | .244^**^ | .172^*^ | .115 | .118 | 1 | .070 | .151^*^ | .165^*^ | .421^**^ |
| **IS** | .279^**^ | .260^**^ | .150^*^ | .153^*^ | .052 | .395^**^ | .338^**^ | .504^**^ | .070 | 1 | .126 | .231^**^ | .047 |
| **TF** | .216^**^ | .094 | .191^**^ | .023 | .102 | -.009 | .087 | .047 | .151^*^ | .126 | 1 | .353^**^ | .266^**^ |
| **RS** | .251^**^ | .074 | .187^**^ | .024 | .230^**^ | -.010 | .057 | .017 | .165^*^ | .231^**^ | .353^**^ | 1 | .196^**^ |
| **HCM** | .382^**^ | .302^**^ | .337^**^ | .216^**^ | .367^**^ | .292^**^ | .292^**^ | .236^**^ | .421^**^ | .047 | .266^**^ | .196^**^ | 1 |

**Notes:** See Supplementary Table S1 for subscale abbreviations; these are Pearson correlations; pairwise deletion was used for missing subscale scores; **Correlation is significant at the 0.01 level (2-tailed); *Correlation is significant at the 0.05 level (2-tailed).

**Supplementary Figure S1: Scree plot and parallel analysis based on sample eigenvalues**


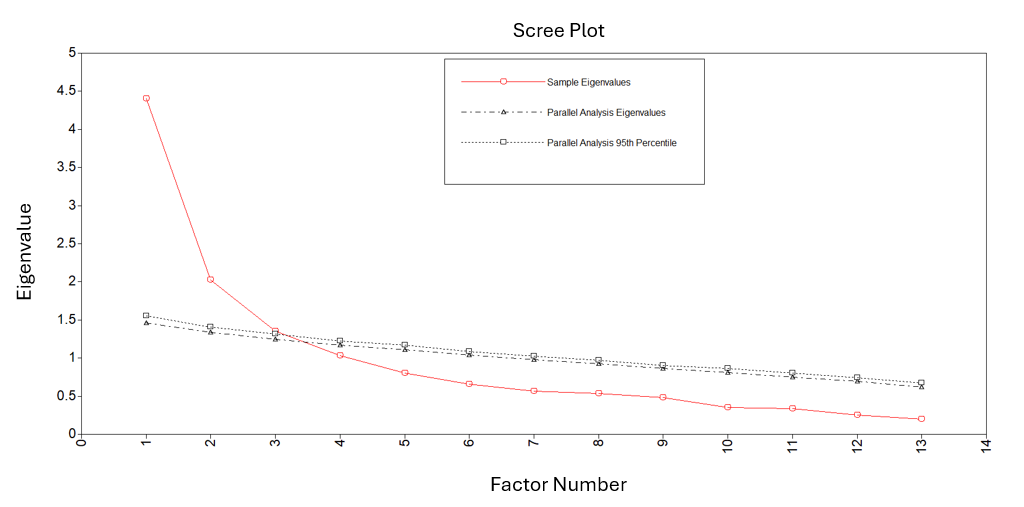


**Supplementary Table S3: Standardized factor loadings and fit statistics from item-level CFAs of the 13 subscales for the CSQ-SCD (Tables S3a – S3m)**

**Note:** For each of the 13 CSQ-SCD subscales, a separate confirmatory factor analysis (CFA) was conducted on the six items comprising each subscale. Because the items are ordered categorical variables with limited response options, the CFAs were conducted using robust weighted least squares estimation (i.e., the WLSMV estimator in Mplus). Below are the results of the 13 CFAs.

*The item numbers refer to the CSQ-SCD questionnaire developed by Gil et al. (1989)

**Table S3a: CFA of Subscale – Diverting Attention**

| **Item Number*** | **Standardized Factor Loadings (SE)** |
| --- | --- |
| Item 4 | 0.817 (0.033) |
| Item 10 | 0.744 (0.047) |
| Item 28 | 0.533 (0.060) |
| Item 34 | 0.561 (0.059) |
| Item 36 | 0.755 (0.036) |
| Item 61 | 0.775 (0.039) |

CFI: 0.975; TLI: 0.958; RMSEA: 0.110; SRMR: 0.028

**Table S3b: CFA of Subscale – Reinterpreting Pain Sensations**

| **Item Number*** | **Standardized Factor Loadings (SE)** |
| --- | --- |
| Item 2 | 0.726 (0.049) |
| Item 31 | 0.717 (0.041) |
| Item 47 | 0.739 (0.038) |
| Item 56 | 0.832 (0.037) |
| Item 63 | 0.813 (0.037) |
| Item 78 | 0.641 (0.054) |

CFI: 0.998; TLI: 0.996; RMSEA: 0.037; SRMR: 0.018

**Table S3c: CFA of Subscale – Calming Self-Statements**

| **Item Number*** | **Standardized Factor Loadings (SE)** |
| --- | --- |
| Item 37 | 0.682 (0.042) |
| Item 39 | 0.581 (0.055) |
| Item 45 | 0.707 (0.039) |
| Item 72 | 0.607 (0.049) |
| Item 73 | 0.772 (0.043) |
| Item 75 | 0.553 (0.060) |

CFI: 0.972; TLI: 0.954; RMSEA: 0.107; SRMR: 0.030

**Table S3d: CFA of Subscale – Ignoring Pain Sensations**

| **Item Number*** | **Standardized Factor Loadings (SE)** |
| --- | --- |
| Item 14 | 0.321 (0.066) |
| Item 41 | 0.749 (0.042) |
| Item 44 | 0.603 (0.049) |
| Item 49 | 0.766 (0.044) |
| Item 58 | 0.673 (0.053) |
| Item 68 | 0.625 (0.057) |

CFI: 0.918; TLI: 0.863; RMSEA: 0.163; SRMR: 0.049

**Table S3e: CFA of Subscale – Praying and Hoping**

| **Item Number*** | **Standardized Factor Loadings (SE)** |
| --- | --- |
| Item 5 | 0.309 (0.075) |
| Item 27 | 0.809 (0.039) |
| Item 42 | 0.331 (0.073) |
| Item 59 | 0.904 (0.038) |
| Item 67 | 0.268 (0.073) |
| Item 77 | 0.866 (0.042) |

CFI: 0.935; TLI: 0.891; RMSEA: 0.157; SRMR: 0.057

**Table S3f: CFA of Subscale – Catastrophizing**

| **Item Number*** | **Standardized Factor Loadings (SE)** |
| --- | --- |
| Item 15 | 0.651 (0.052) |
| Item 21 | 0.768 (0.035) |
| Item 46 | 0.752 (0.038) |
| Item 57 | 0.807 (0.039) |
| Item 60 | 0.832 (0.032) |
| Item 76 | 0.685 (0.055) |

CFI: 0.967; TLI: 0.944; RMSEA: 0.145; SRMR: 0.035

**Table S3g: CFA of Subscale – Fear Self-Statements**

| **Item Number*** | **Standardized Factor Loadings (SE)** |
| --- | --- |
| Item 20 | 0.652 (0.049) |
| Item 29 | 0.827 (0.033) |
| Item 51 | 0.779 (0.036) |
| Item 54 | 0.804 (0.039) |
| Item 65 | 0.523 (0.059) |
| Item 71 | 0.605 (0.052) |

CFI: 0.991; TLI: 0.986; RMSEA: 0.064; SRMR: 0.025

**Table S3h: CFA of Subscale – Anger Self-Statements**

| **Item Number*** | **Standardized Factor Loadings (SE)** |
| --- | --- |
| Item 8 | 0.747 (0.038) |
| Item 17 | 0.747 (0.041) |
| Item 23 | 0.751 (0.040) |
| Item 38 | 0.418 (0.078) |
| Item 43 | 0.763 (0.041) |
| Item 48 | 0.672 (0.047) |

CFI: 0.896; TLI: 0.827; RMSEA: 0.223; SRMR: 0.071

**Table S3i: CFA of Subscale – Increasing Behavioral Activities**

| **Item Number*** | **Standardized Factor Loadings (SE)** |
| --- | --- |
| Item 6 | 0.339 (0.082) |
| Item 18 | 0.372 (0.081) |
| Item 40 | 0.240 (0.078) |
| Item 53 | 0.335 (0.072) |
| Item 62 | 0.676 (0.069) |
| Item 69 | 0.837 (0.079) |

CFI: 0.728; TLI: 0.547; RMSEA: 0.184; SRMR: 0.061

**Table S3j: CFA of Subscale – Isolation**

| **Item Number*** | **Standardized Factor Loadings (SE)** |
| --- | --- |
| Item 7 | 0.574 (0.051) |
| Item 9 | 0.655 (0.046) |
| Item 24 | 0.695 (0.042) |
| Item 26 | 0.890 (0.026) |
| Item 30 | 0.787 (0.033) |
| Item 35 | 0.656 (0.043) |

CFI: 0.970; TLI: 0.950; RMSEA: 0.131; SRMR: 0.033

**Table S3k: CFA of Subscale – Taking Fluids**

| **Item Number*** | **Standardized Factor Loadings (SE)** |
| --- | --- |
| Item 11 | 0.407 (0.058) |
| Item 13 | 0.851 (0.033) |
| Item 16 | 0.710 (0.041) |
| Item 19 | 0.901 (0.029) |
| Item 50 | 0.734 (0.044) |
| Item 64 | 0.824 (0.036) |

CFI: 0.986; TLI: 0.977; RMSEA: 0.099; SRMR: 0.030

**Table S3l: CFA of Subscale – Resting**

| **Item Number*** | **Standardized Factor Loadings (SE)** |
| --- | --- |
| Item 1 | 0.493 (0.054) |
| Item 25 | 0.676 (0.052) |
| Item 52 | 0.456 (0.061) |
| Item 55 | 0.683 (0.049) |
| Item 66 | 0.775 (0.045) |
| Item 70 | 0.775 (0.045) |

CFI: 0.887; TLI: 0.811; RMSEA: 0.200; SRMR: 0.062

**Table S3m: CFA of Subscale – Heat/Cold/Massage**

| **Item Number*** | **Standardized Factor Loadings (SE)** |
| --- | --- |
| Item 3 | 0.838 (0.039) |
| Item 12 | 0.850 (0.043) |
| Item 22 | 0.828 (0.037) |
| Item 32 | 0.789 (0.041) |
| Item 33 | 0.168 (0.076) |
| Item 74 | 0.366 (0.064) |

CFI: 0.878; TLI: 0.797; RMSEA: 0.296; SRMR: 0.106

**Supplementary Table S4: Item means, standard deviations, and correlations (Pearson and polychoric) for the CSQ-SCD (Tables S4a – S4c)**

Note: The item numbers (i.e., C1-C78) refer to items in CSQ-SCD questionnaire developed by Gil et al. (1989)

**Table S4a: Item means and standard deviations**

| **Items** | **Mean** | **Std. Deviation** | **N** |
| --- | --- | --- | --- |
| C1 | 3.72 | 1.662 | 194 |
| C2 | 1.37 | 1.746 | 193 |
| C3 | 4.00 | 1.817 | 196 |
| C4 | 3.52 | 1.891 | 194 |
| C5 | 2.84 | 2.257 | 193 |
| C6 | 2.55 | 1.959 | 195 |
| C7 | 3.88 | 1.594 | 195 |
| C8 | 2.95 | 1.880 | 196 |
| C9 | 2.82 | 1.700 | 195 |
| C10 | 4.54 | 1.557 | 195 |
| C11 | 3.77 | 2.094 | 195 |
| C12 | 5.20 | 1.344 | 194 |
| C13 | 5.22 | 1.105 | 196 |
| C14 | 2.56 | 1.904 | 194 |
| C15 | 4.04 | 1.611 | 195 |
| C16 | 4.44 | 1.637 | 196 |
| C17 | 3.66 | 1.973 | 194 |
| C18 | 4.77 | 1.486 | 195 |
| C19 | 5.17 | 1.292 | 196 |
| C20 | 2.09 | 1.731 | 195 |
| C21 | 3.03 | 1.786 | 191 |
| C22 | 4.23 | 1.840 | 192 |
| C23 | 3.40 | 1.822 | 192 |
| C24 | 3.60 | 1.757 | 192 |
| C25 | 4.17 | 1.525 | 193 |
| C26 | 3.89 | 1.679 | 192 |
| C27 | 4.99 | 1.623 | 193 |
| C28 | 2.57 | 2.068 | 191 |
| C29 | 3.47 | 1.777 | 193 |
| C30 | 2.86 | 1.806 | 192 |
| C31 | 1.74 | 1.759 | 192 |
| C32 | 4.84 | 1.471 | 192 |
| C33 | 1.07 | 1.777 | 193 |
| C34 | 2.91 | 1.911 | 193 |
| C35 | 3.90 | 1.708 | 193 |
| C36 | 2.97 | 1.968 | 192 |
| C37 | 4.49 | 1.410 | 191 |
| C38 | .35 | 1.104 | 191 |
| C39 | 4.83 | 1.423 | 191 |
| C40 | 2.15 | 1.690 | 190 |
| C41 | 2.51 | 1.701 | 188 |
| C42 | 3.44 | 1.878 | 190 |
| C43 | 3.83 | 1.896 | 191 |
| C44 | 2.82 | 1.752 | 190 |
| C45 | 4.45 | 1.457 | 191 |
| C46 | 3.23 | 1.927 | 191 |
| C47 | 1.39 | 1.641 | 191 |
| C48 | 4.03 | 1.746 | 188 |
| C49 | 1.86 | 1.584 | 191 |
| C50 | 4.80 | 1.516 | 191 |
| C51 | 3.70 | 1.754 | 184 |
| C52 | 3.71 | 1.463 | 185 |
| C53 | 4.59 | 1.345 | 185 |
| C54 | 2.85 | 2.059 | 184 |
| C55 | 5.10 | 1.143 | 185 |
| C56 | 1.63 | 1.777 | 185 |
| C57 | 1.72 | 1.670 | 185 |
| C58 | 1.92 | 1.781 | 185 |
| C59 | 5.13 | 1.519 | 184 |
| C60 | 3.20 | 1.796 | 185 |
| C61 | 3.51 | 1.823 | 182 |
| C62 | 2.44 | 1.893 | 182 |
| C63 | 1.41 | 1.740 | 182 |
| C64 | 5.04 | 1.337 | 181 |
| C65 | 4.10 | 1.502 | 181 |
| C66 | 5.03 | 1.201 | 181 |
| C67 | 3.23 | 1.775 | 182 |
| C68 | 2.76 | 1.657 | 182 |
| C69 | 1.63 | 1.761 | 181 |
| C70 | 5.25 | 1.072 | 180 |
| C71 | 3.18 | 1.781 | 181 |
| C72 | 4.21 | 1.535 | 181 |
| C73 | 4.00 | 1.538 | 181 |
| C74 | 5.04 | 1.324 | 181 |
| C75 | 2.71 | 1.803 | 181 |
| C76 | 1.24 | 1.706 | 180 |
| C77 | 5.21 | 1.374 | 180 |
| C78 | .95 | 1.415 | 181 |

**Table S4b: Pearson correlations among the items (pairwise deletion was used for missing item scores)**


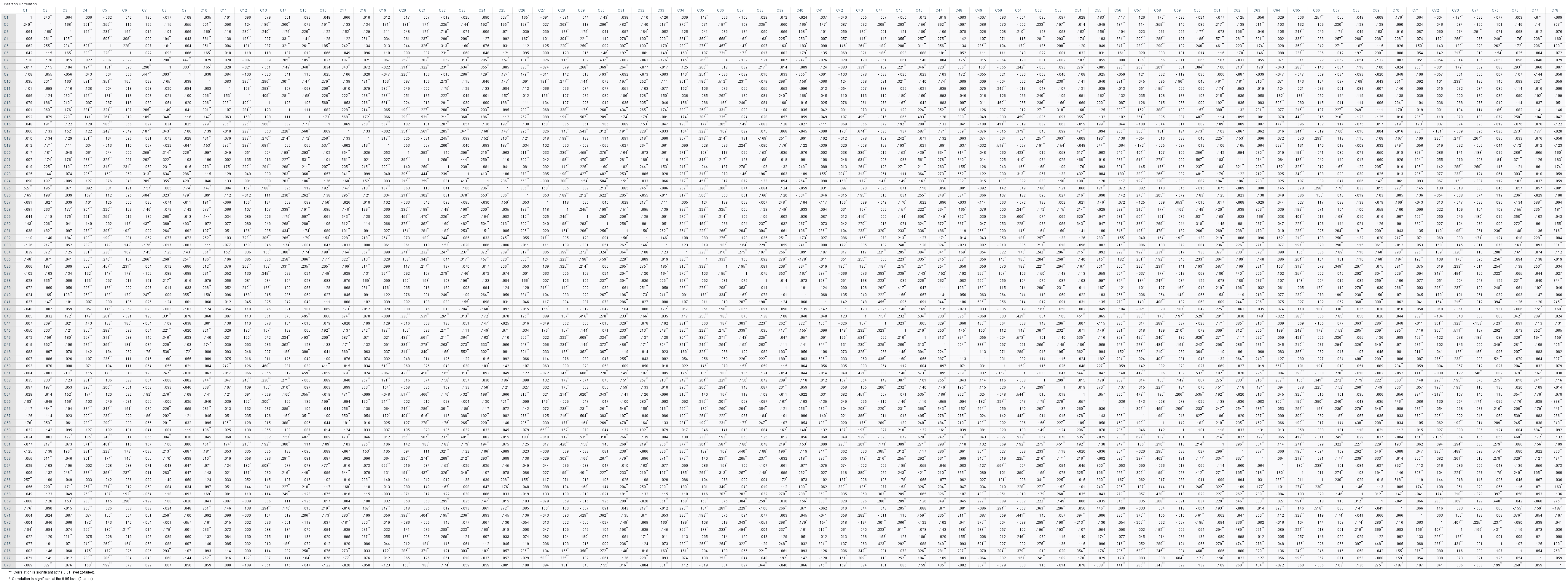


**Table S4c: Polychoric correlations among the items (pairwise deletion was used for missing item scores)**


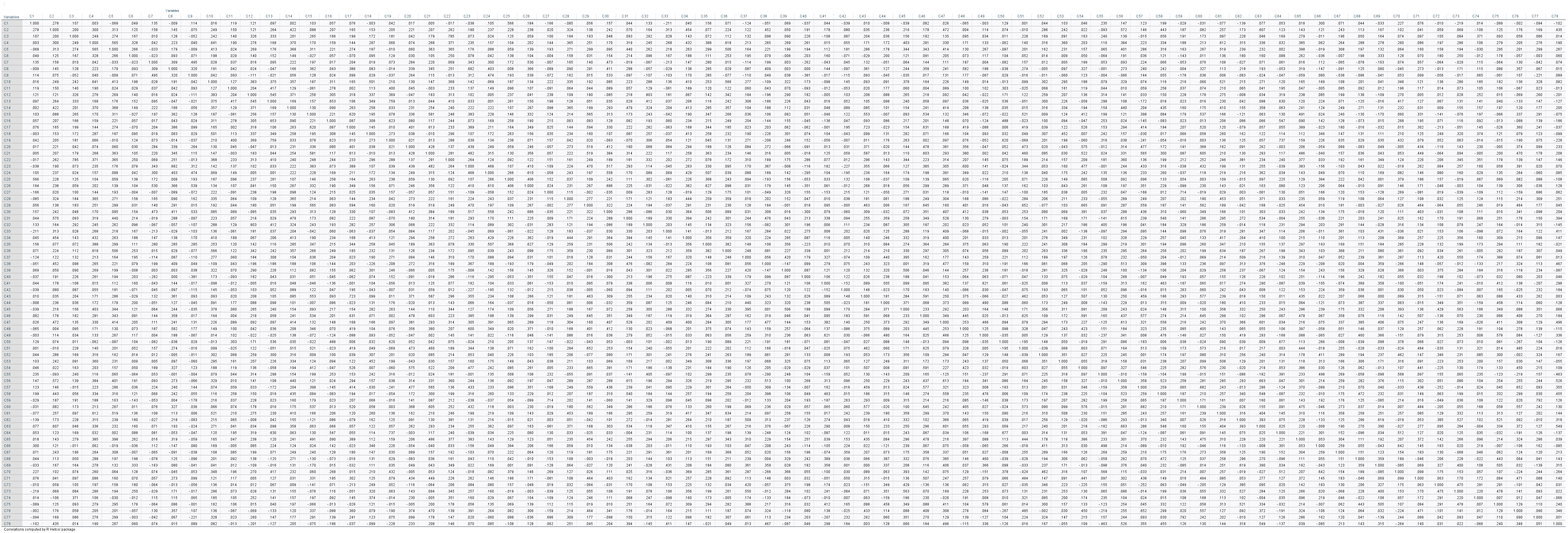


**MPlus and SPSS Code**

Mplus code for Table 2 in the manuscript: Summary of model fit indices for the CSQ-SCD confirmatory factor models

AND

Mplus code for Table 3 in the manuscript: Standardized factor loadings for the final three-factor model of coping for the CSQ-SCD among adults with SCD

TITLE: CFA of 3-FACTOR MODEL OF CSQ-SCD

DATA: FILE IS XYZ;

VARIABLE: NAMES ARE !Names of variables have been fully

spelled out rather than the

abbreviated names used in Mplus

C1-C78

average_diverting_attention

average_reinterpreting_pain_sensations

average_calming_self_statements

average_ignoring_pain_sensations

average_praying_hoping

average_catastrophizing

average_fear_self_statements

average_anger_self_statements

average_increasing_behavioral_activity

average_isolation

average_taking_fluids

average_resting

average_heat_cold_massage;

USEVARIABLES ARE

average_diverting_attention

average_reinterpreting_pain_sensations

average_calming_self_statements

average_ignoring_pain_sensations

average_praying_hoping

average_catastrophizing

average_fear_self_statements

average_anger_self_statements

average_increasing_behavioral_activity

average_isolation

average_taking_fluids

average_resting

average_heat_cold_massage;

MISSING ARE ALL (-99);

ANALYSIS: ESTIMATOR=ML;

MODEL:

ACTIVE_COPING BY average_diverting_attention

average_reinterpreting_pain_sensations

average_calming_self_statements

average_ignoring_pain_sensations

average_increasing_behavioral_activity;

AFFECTIVE_COPING BY average_praying_hoping

average_catastrophizing

average_fear_self_statements

average_anger_self_statements

average_isolation;

PASSIVE_COPING BY average_resting

average_taking_fluids

average_heat_cold_massage;

OUTPUT: SAMPSTAT STAND TECH4;

TITLE: CFA of 2-FACTOR MODEL OF CSQ-SCD

DATA: FILE IS XYZ;

VARIABLE: NAMES ARE !Names of variables have been fully

spelled out rather than the

abbreviated names used in Mplus

C1-C78

average_diverting_attention

average_reinterpreting_pain_sensations

average_calming_self_statements

average_ignoring_pain_sensations

average_praying_hoping

average_catastrophizing

average_fear_self_statements

average_anger_self_statements

average_increasing_behavioral_activity

average_isolation

average_taking_fluids

average_resting

average_heat_cold_massage;

USEVARIABLES ARE

average_diverting_attention

average_reinterpreting_pain_sensations

average_calming_self_statements

average_ignoring_pain_sensations

average_praying_hoping

average_catastrophizing

average_fear_self_statements

average_anger_self_statements

average_increasing_behavioral_activity

average_isolation

average_taking_fluids

average_resting

average_heat_cold_massage;

MISSING ARE ALL (-99);

ANALYSIS: ESTIMATOR=ML;

MODEL:

COPING_ATTEMPTS BY average_diverting_attention

average_reinterpreting_pain_sensations

average_calming_self_statements

average_ignoring_pain_sensations

average_praying_hoping

average_increasing_behavioral_activity;

NEGATIVE_THINKING BY average_catastrophizing

average_fear_self_statements

average_anger_self_statements

average_isolation

average_taking_fluids

average_resting

average_heat_cold_massage;

OUTPUT: SAMPSTAT STAND TECH4;

Mplus code for Table 4 in the manuscript: Discriminant validity (i.e., tests of perfect correlations) assessed by Wald’s Chi-square test

TITLE: DISCRIMINANT VALIDITY OF 3-FACTOR CSQ-SCD

DATA: FILE IS XYZ;

VARIABLE: NAMES ARE !Names of variables have been fully

spelled out rather than the

abbreviated names used in Mplus

C1-C78

average_diverting_attention

average_reinterpreting_pain_sensations

average_calming_self_statements

average_ignoring_pain_sensations

average_praying_hoping

average_catastrophizing

average_fear_self_statements

average_anger_self_statements

average_increasing_behavioral_activity

average_isolation

average_taking_fluids

average_resting

average_heat_cold_massage;

USEVARIABLES ARE

average_diverting_attention

average_reinterpreting_pain_sensations

average_calming_self_statements

average_ignoring_pain_sensations

average_praying_hoping

average_catastrophizing

average_fear_self_statements

average_anger_self_statements

average_increasing_behavioral_activity

average_isolation

average_taking_fluids

average_resting

average_heat_cold_massage;

MISSING ARE ALL (-99);

ANALYSIS: ESTIMATOR=ML;

ITERATIONS=5000;

MODEL:

ACTIVE_COPING BY average_diverting_attention*

average_reinterpreting_pain_sensations

average_calming_self_statements

average_ignoring_pain_sensations

average_increasing_behavioral_activity;

AFFECTIVE_COPING BY average_praying_hoping*

average_catastrophizing

average_fear_self_statements

average_anger_self_statements

average_isolation;

PASSIVE_COPING BY average_resting*

average_taking_fluids

average_heat_cold_massage;

ACTIVE_COPING WITH AFFECTIVE_COPING (a1);

AFFECTIVE_COPING WITH PASSIVE_COPING (a2);

ACTIVE_COPING WITH PASSIVE_COPING (a3);

ACTIVE_COPING@1;

AFFECTIVE_COPING@1;

PASSIVE_COPING@1;

MODEL TEST: a1=1; !test for a2 and a3 as well;

SPSS code for Table 7 in the manuscript: Varimax rotated factor loadings for three-factor exploratory factor analysis model using principal factors extraction

*Names of variables have been fully spelled out rather than the abbreviated names used in SPSS.

FACTOR

/VARIABLES average_diverting_attention

average_reinterpreting_pain_sensations average_calming_self_statements average_ignoring_pain_sensations average_praying_hoping average_catastrophizing average_fear_self_statements average_anger_self_statements average_increasing_behavioral_activity average_isolation average_taking_fluids average_resting average_heat_cold_massage

/MISSING LISTWISE

/ANALYSIS average_diverting_attention average_reinterpreting_pain_sensations average_calming_self_statements average_ignoring_pain_sensations average_praying_hoping average_catastrophizing average_fear_self_statements average_anger_self_statements average_increasing_behavioral_activity average_isolation average_taking_fluids average_resting average_heat_cold_massage

/PRINT INITIAL EXTRACTION ROTATION

/CRITERIA FACTORS(3) ITERATE(25)

/EXTRACTION PAF

/CRITERIA ITERATE(25)

/ROTATION VARIMAX

/METHOD=CORRELATION.
